# Matrine prevents bone loss in ovariectomized mice by inhibiting RANKL-induced osteoclastogenesis

**DOI:** 10.1096/fj.201700316R

**Published:** 2017-07-24

**Authors:** Xiao Chen, Xin Zhi, Panpan Pan, Jin Cui, Liehu Cao, Weizong Weng, Qirong Zhou, Lin Wang, Xiao Zhai, Qingiie Zhao, Honggang Hu, Biaotong Huang, Jiacan Su

**Affiliations:** * Department of Orthopedics Trauma and Shanghai Changhai Hospital, Second Military Medical University, Shanghai, China;; † China–South Korea Bioengineering Center, Shanghai, China;; ‡ Graduate Management Unit, Shanghai Changhai Hospital, Second Military Medical University, Shanghai, China; and; § School of Pharmacy, Second Military Medical University, Shanghai, China

**Keywords:** postmenopausal osteoporosis, NFATc1, osteoclasts

## Abstract

Osteoporosis is a metabolic bone disease characterized by decreased bone density and strength due to excessive loss of bone protein and mineral content. The imbalance between osteogenesis by osteoblasts and osteoclastogenesis by osteoclasts contributes to the pathogenesis of postmenopausal osteoporosis. Estrogen withdrawal leads to increased levels of proinflammatory cytokines. Overactivated osteoclasts by inflammation play a vital role in the imbalance. Matrine is an alkaloid found in plants from the *Sophora* genus with various pharmacological effects, including anti-inflammatory activity. Here we demonstrate that matrine significantly prevented ovariectomy-induced bone loss and inhibited osteoclastogenesis *in vivo* with decreased serum levels of TRAcp5b, TNF-α, and IL-6. *In vitro* matrine significantly inhibited osteoclast differentiation induced by receptor activator for NF-κB ligand (RANKL) and M-CSF in bone marrow monocytes and RAW264.7 cells as demonstrated by tartrate-resistant acid phosphatase (TRAP) staining and actin-ring formation as well as bone resorption through pit formation assays. For molecular mechanisms, matrine abrogated RANKL-induced activation of NF-κB, AKT, and MAPK pathways and suppressed osteoclastogenesis-related marker expression, including matrix metalloproteinase 9, NFATc1, TRAP, C-Src, and cathepsin K. Our study demonstrates that matrine inhibits osteoclastogenesis through modulation of multiple pathways and that matrine is a promising agent in the treatment of osteoclast-related diseases such as osteoporosis.—Chen, X., Zhi, X., Pan, P., Cui, J., Cao, L., Weng, W., Zhou, Q., Wang, L., Zhai, X. Zhao, Q., Hu, H., Huang, B., Su, J. Matrine prevents bone loss in ovariectomized mice by inhibiting RANKL-induced osteoclastogenesis.

Osteoporosis is a metabolic bone disease characterized by decreased bone density and strength due to excessive loss of bone protein and mineral content (1). Postmenopausal osteoporosis (PMOP) is the most common form of primary osteoporosis, and the incidence of PMOP has been increasing rapidly (2). PMOP leads to increased risk of osteoporotic fractures.

The imbalance between osteogenesis by osteoblasts and osteoclastogenesis by osteoclasts contributes to the pathogenesis of PMOP (3). Estrogen is osteoprotective. On one hand, it targets osteoblasts and stimulates osteoblasts to secrete OPG, which could rival with receptor activator for NF-κB ligand (RANKL) and inhibit osteoclastogenesis (4, 5). On the other hand, estrogen directly inhibits preosteoclast differentiation and osteoclast formation and function (6) and induces apoptosis of osteoclasts and preosteoclasts to reduce the number of osteoclasts (7). After menopause, withdrawal of estrogen leads to an increase of osteoclastogenesis and a decrease of osteogenesis. Thus, postmenopausal osteoporosis occurs. Osteoclasts are bone-resorbing multinucleated giant cells differentiated from hematopoietic precursor cells of monocyte–macrophage lineage (8). Osteoclasts overactivated by inflammation play a vital role in the imbalance (9). Thus, inhibiting osteoclastognenesis through suppressing inflammation is an important strategy for preventing and treating PMOP (10–12). RANKL is an essential factor for osteoclast differentiation and function. The conjunct of RANKL and RANK recruits TNF receptor–associated factors (TRAFs), typically TRAF6 (13), which activates multiple downstream signaling pathways, mainly NF-κB, MAPKs, and AKT, and thereby initiates osteoclast differentiation and bone resorption by inducing transcription and expression of osteoclast-specific genes, such as tartrate-resistant acid phosphatase (TRAP), cathepsin K, matrix metalloproteinase 9 (MMP-9), and C-Src (14).

Matrine is the major component of the traditional Chinese herb *Sophora flavescens* Ait. Studies have demonstrated a number of pharmacological effects of matrine, such as its anti-tumor and anti-inflammation effects (15–17), and it has been widely used clinically against hepatitis because of its anti-inflammatory effects (18). Because inflammation is closely related to PMOP, we hypothesized that matrine could possess antiosteoporotic effects and serve as a promising candidate for antiosteoporosis drug development. We carried out this study to investigate the effects of matrine on ovariectomy-induced bone loss and to explore the possible underlying molecular mechanisms.

## MATERIALS AND METHODS

### Reagents and antibodies

**Figure 1.**
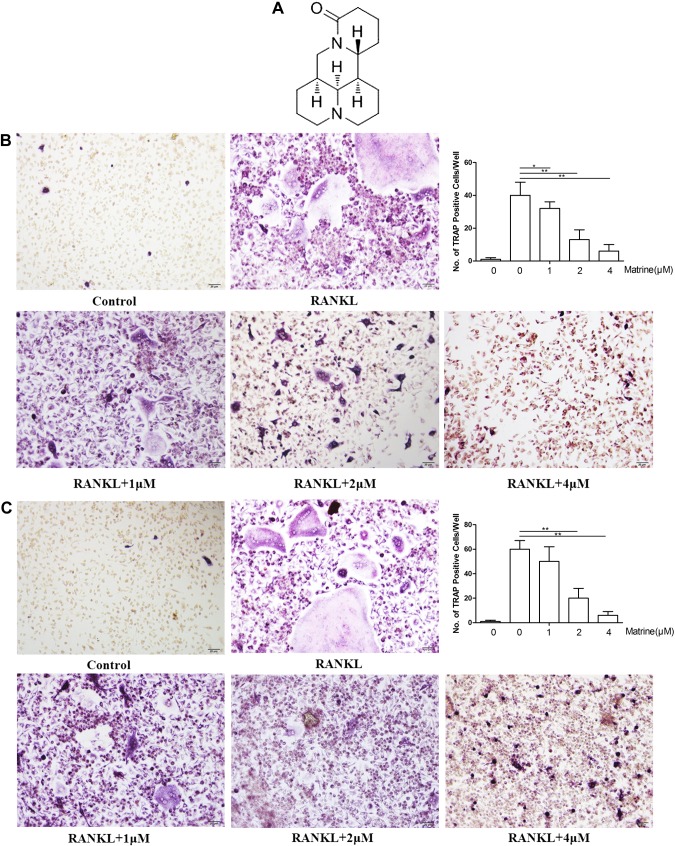
Matrine inhibits osteoclastogenesis *in vitro*. *A*) Chemical structure of matrine. *B*) Formation of TRAP-positive cells from BMMCs and quantification of osteoclasts. *C*) Formation of TRAP-positive cells from RAW264.7 cells and quantification of osteoclasts. *P < 0.05, **P < 0.01.

Matrine (**Fig. 1*A***) was provided by H.H. It was dissolved in PBS (vehicle) for further use. RAW 264.7 cells were obtained from Prof. J. Hou (Second Military Medical University). Penicillin, streptomycin, and fetal bovine serum were obtained from Puhe Biotechnology Co. (Wu, China).

### Animals and experimental design

All experiments were performed in the Specific Pathogen Free laboratory of Shanghai Changhai Hospital. Female, 8-wk-old, C57BL/6 mice were purchased from Shanghai Slack Co. (Shanghai, China) and kept under standard conditions with free access to clean water and food. All procedures were in accordance with the guidelines of the Ethics Committee on Animal Experiment of the Second Military Medical University. Animals were randomly assigned to 3 groups (*n* = 6/group): a sham-treated group, ovariectomized (OVX) mice treated with normal saline, and OVX mice treated with matrine dissolved in normal saline. The mice in the OVX group were anesthetized with 5% chloral hydrate. Then, small incisions were made on the dorsal skin and peritoneum. Two ovaries and part of the oviduct were removed and pressed to stop any bleeding. The incision on the skin was closed with 5-0 nonabsorbable suture lines. After the procedure, mice were allowed to recover for 24 h. From the second postoperative day, 150 mg/kg/d of matrine or normal saline was given by intraperitoneal injection. After 6 wk of intervention, all mice were anesthetized with chloral hydrate, and the femur and arterial blood was obtained. No significant adverse effects were observed after matrine was administered.

### *In vitro* osteoclastogenesis assay

Bone marrow monocytes (BMMs) were obtained from the femoral bone marrow of C57BL/6 mice at 8 wk of age. BMMs and RAW264.7 cells were seeded (8 × 10^3^ cells/well) onto 24-well plates with DMEM low-glucose medium with 10% heat-inactivated fetal bovine serum and a penicillin (100 U/ml)/streptomycin (100 mg/ml) mixture and incubated. Cells were cultured in cell culture medium at 37°C and 5% CO_2_. Nonadherent cells were removed by frequent medium changes over 72 h. The remaining adherent colonies were cultured for 14 d until confluent and passaged after digestion with 0.25% trypsin for 3 min and subcultured. The third-generation BMMs (2.5 × 10^3^ cells/well) and RAW264.7 cells (1.5 × 10^3^ cells/well) were cultured on 96-well plates and divided into a control group and 4 groups treated with matrine (0, 1, 2, or 4 μM). The matrine-treated cells were induced into osteoclasts by M-CSF (20 ng/ml) and RANKL (50 ng/ml). On d 7, the BMMs of the control group and the matrine-treated groups were stained by tartrate-resistant acid phosphatase (TRAP) using a TRAP staining kit (Sigma-Aldrich, St. Louis, MO, USA) according to the manufacturer’s protocol. More than 3 nucleus cells were regarded as osteoclast cells and counted. Cells were cultured for 24 h and fixed with 4% paraformaldehyde in PBS for 10 min. The cells were permeabilized with 0.1% Triton-X 100 in PBS for 5 min and incubated with rhodamine-conjugated phalloidin (Biotium, Fremont, CA, USA) to visualize F-actin. All experiments were carried out 3 times, and the average was calculated.

### Pit-formation assays

RAW264.7 cells (1.5 × 10^3^ cells/well) were seeded on bone biomimetic synthetic surface (Osteo Assay Surface 24-Well Multiple Well Plates; Corning, Corning, NY, USA) in the absence or presence of 100 ng/ml RANKL with or without varying concentrations of matrine (1, 2, or 4 μM) and incubated. Medium was changed on d 3 as reported previously (11). After 7 d, the plates were washed with PBS and air dried for 3–5 h. The osteoclast-resorbing area was captured using a light microscope (BX53; Olympus, Tokyo, Japan). The resorbed area was quantified using Image-Pro Plus software.

### Immunofluorescence staining

The effects of matrine on the nuclear translocation of P65 were determined by immunofluorescence as previously described (19, 20). The BMMs of the control group and BMMs treated with matrine (0 or 4 μM) were fixed with 4% paraformaldehyde for 15 min, washed with 0.2% Triton X-100 in PBS for 10 min, blocked with 1% BSA in PBS, and incubated with monoclonal anti-P65 antibody (Abcam, Cambridge, MA, USA) followed by biotinylated goat anti-mouse IgG antibody (Abcam) and fluorescein-conjugated streptavidin (Vector Laboratories, Burlingame, CA, USA). Cells were counterstained with propidium iodide (Vector Laboratories).

### Western blotting

The effects of matrine on NF-κB, MAPKs, and AKT pathways in RAW264.7 cells were evaluated by Western blotting. The RAW264.7 cells were seeded (2 × 10^6^ cells/well) into 6-well plates and divided into 2 groups: cells treated with RANKL and cells treated with matrine (0 or 4 μM). Cells were evaluated by Western blotting at 0, 15, 30, 45, and 60 min to observe phosphorylation of IκB, P65, P50, ERK, JNK, P38, C-fos, and AKT. The 3 groups of RAW264.7 cells (control, treated with RANKL and matrine 0 or 4 μM) were cultured in 6-well plates (2 × 10^6^ cells/well) for 7 d and measured by Western blotting. At d 7, the expression levels of osteoclastogenesis-related markers MMP-9, TRAP, cathepsin K, C-Src, and NFATc1 were determined. The primary antibodies included mouse anti-GAPDH and mouse anti–β-actin. Antibodies specific to P-ERK, ERK, P-JNK, JNK, P-P38, P38, P-C-fos, C-fos, P-AKT, AKT, P-IκB, IκB, P-P65, P65, P-P50, P50, MMP-9, cathepsin K, C-Src, TRAP, NFATc1, and β-actin were supplied by Abcam. Secondary antibodies included goat anti-rabbit IgG–horseradish peroxidase (Abcam) and donkey anti-goat horseradish peroxidase–HRP (Abcam).

### Bone histomorphometric analysis

Femurs were fixed in 4% formalin for 4 d and decalcified for 2 wk using 10% tetrasodium-EDTA aqueous solution. Sections (4 μm thick) were prepared with a microtome (Jung, Heidelberg, Germany) and stained with hematoxylin and eosin (H&E). Osteoclasts were visualized by TRAP staining. Standard bone histomorphometric measures were analyzed by a microscope (original magnification, ×40) (BX53; Olympus). Trabecular bone was revealed in H&E-stained sections, and the red box area (0.498 mm^2^) was monitored by Image-Pro Plus.

### Microcomputed tomography analysis

The femur was analyzed by micro–computed tomography (Skyscan, Antwerp, Belgium). The following acquisition parameters were used: 80 kV, 124 μA, voxel size in reconstructed image, 8 μm. Images were analyzed using a plug-in programmed with the following histomorphometric parameters at the metaphysis of the proximal tibiae: bone volume/total volume (BV/TV), bone surface area/total volume (BS/TV), bone mineral density (BMD), and trabecular number. Two-dimensional and 3-dimensional bone structure images were published by the built-in software.

### Serum biochemistry

Blood was collected *via* heart puncture, and sera were collected after centrifugation at 3000 rpm for 15 min at 25°C. Serum levels of IL-6, TNF-α, TRAcp5b, and osteocalcin (OCN) were measured with an ELISA kit (Anogen, Mississauga, ON, Canada) according to the manufacturer’s instructions.

### Statistical analysis

All results are expressed as means ± sd. Statistical analysis was performed by 2‐tailed, unpaired Student’s *t* test to compare 2 groups, and by 1-way ANOVA to compare 3 or more groups using SPSS Statistics 21.0 (IBM, Armonk, NY, USA). Values of *P* < 0.05 were considered statistically significant.

## RESULTS

### Matrine inhibits osteoclastogenesis and functions *in vitro*

To examine the effects of matrine on osteoclastogenesis, BMMs were treated with RANKL and M-CSF in the presence of 1-, 2-, and 4-μm concentrations of matrine. The results indicated that osteoclast formation was compromised by matrine in a concentration-dependent manner (**Fig. 1*B*, *C***). The total number of TRAP-positive cells was significantly lower as matrine concentration increased from 1 to 4 μm matrine compared with the control group (*P* < 0.05). Among different matrine-treated groups, the number of TRAP-positive cells in the 1-μm group was significantly less than that in 2- and 4-μm groups (*P* < 0.05). These results suggest that matrine dose-dependently inhibits RANKL-induced osteoclastogenesis in BMMs and RAW264.7 cells.

**Figure 2.**
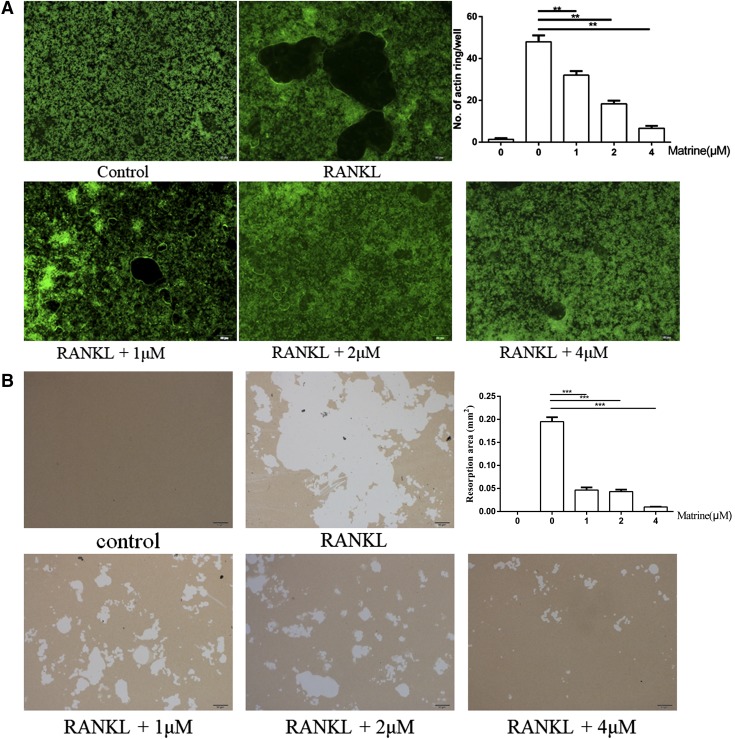
Matrine inhibits osteoclast function *in vitro*. *A*) Actin ring structures of osteoclasts and quantification of the actin ring. *B*) RAW264.7 cells seeded on hydroxyapatite-coated plates. Surfaces were treated similarly and incubated for 7 d, and resorption area was quantified by image analysis. ***P < 0.001.

To determine the functions of osteoclasts, we examined whether matrine affected RANKL-induced osteoclast actin ring formation, which is the most obvious feature of mature osteoclasts during osteoclastogenesis. When incubated with RANKL, RAW264.7 cells differentiated into mature osteoclasts and formed actin rings. However, the size and number of actin ring structures were significantly reduced when the cells were treated with matrine, suggesting that matrine suppressed the formation of actin rings in mature osteoclasts (**Fig. 2*A***). We next examined the effects of matrine on the pit-forming activity on bone biomimetic synthetic surface. Osteoclast activity was impaired severely by matrine treatment, as demonstrated by diminished resorption pits formed by osteoclasts. When the osteoclast preparation was cultured on dentine plates for 24 h, many resorption pits formed on the plates. Matrine added at 1, 2, and 4 μm significantly inhibited the pit-forming activity of osteoclasts (Fig. 2*B*).

### Matrine inhibits RANKL-induced activation of the NF-κB pathway

**Figure 3.**
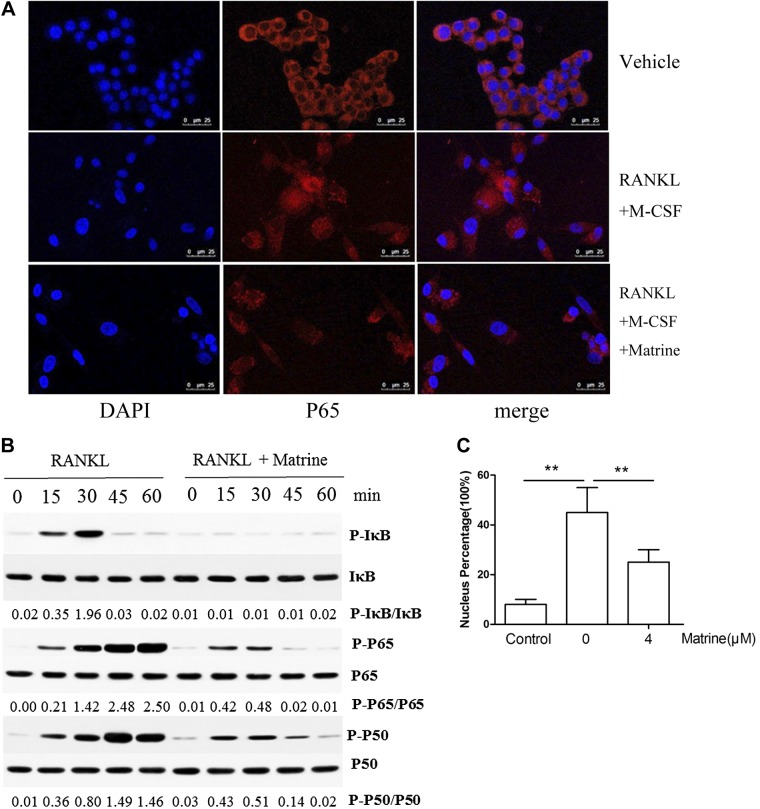
Matrine inhibits RANKL-induced NF-κB activation. *A*) Matrine inhibits RANKL-induced P65 nuclear translocation. *B*) Phosphorylation of P65, P50, and IκB protein, which were associated with the NF-κB pathway. *C*) Ratio of the fluorescence intensity at the nuclear site with whole-cell fluorescence intensity. **P < 0.01.

RANKL-induced NF-κB activation is necessary for osteoclast differentiation and function. To determine whether matrine inhibits NF-κB–mediated osteoclastogenesis, we performed immunofluorescence staining of P65 with or without matrine in RAW264.7 cells. In RAW264.7 cells, immunofluorescence staining showed that most P65 was located in the cytoplasm. After induction with RANKL and M-CSF in RAW264.7 cells, P65 was phosphorylated and active and translocated to the nucleus. However, the nuclear translocation of P65 was blocked when treated with matrine despite induction with RANKL and M-CSF (*P* < 0.05) (**Fig. 3*A*, *B***). To confirm the results of immunofluorescence staining, assays we demonstrated using Western blot assays that matrine could inhibit RANKL-induced phosphorylation and degradation of the inhibitory subunit of NF-κB (IκB) as well as the phosphorylation of P65 and P50 (Fig. 3*C*). The results indicate that matrine can inhibit the NF-κB pathway.

### Marine inhibits RANKL-induced activation of MAPKs and the AKT pathway

**Figure 4.**
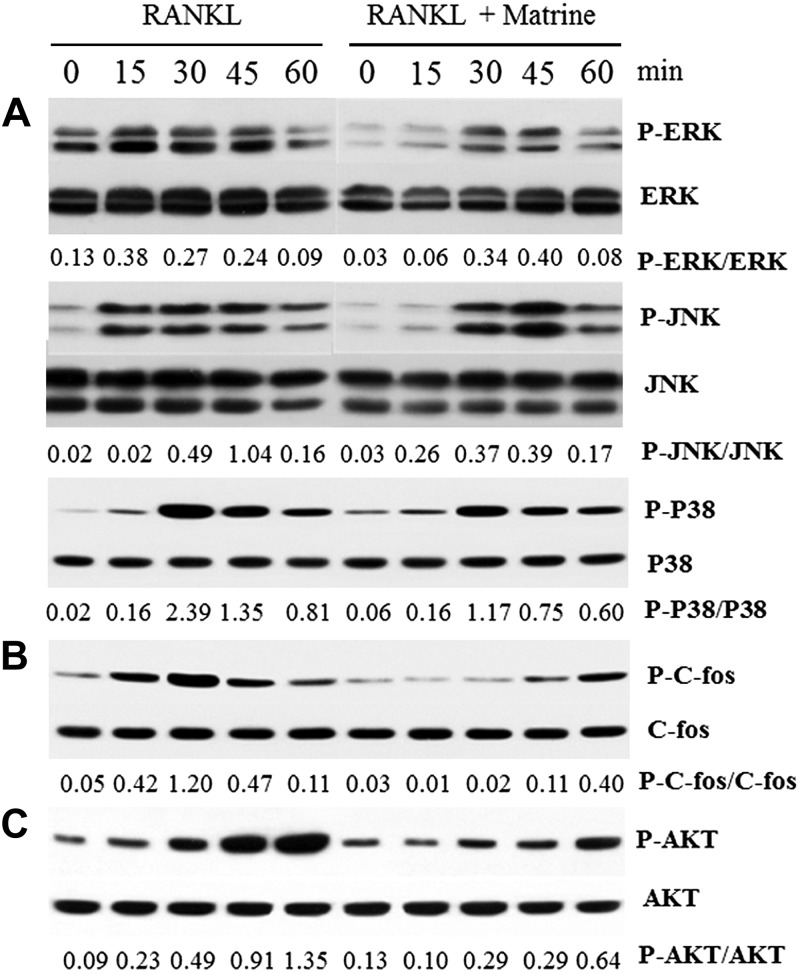
Matrine suppresses MAPKs and PI3K/AKT pathways in osteoclastogenesis. *A*) Phosphorylation of ERK, JNK, and P38, which was associated with the MAPK pathway. *B*) Phosphorylation of C-fos, which was an important downstream transcription factor of the MAPK pathway. *C*) Phosphorylation of AKT, which was associated with the PI3K/AKT pathways.

In addition to the NF-κB signaling pathway, activation of MAPKs and the AKT pathway plays an important role in osteoclastogenesis. To evaluate the effects of matrine on MAPKs and the AKT pathway after incubation with RANKL and RAW264.7 cells, we examined the phosphorylation of 3 major subfamilies of MAPKs [P38 (p-p38), JNK (p-JNK), and ERK (p-ERK)] (**Fig. 4*A***) as well as C-fos (p-c-fos) (Fig. 4*B*) and AKT (p-Akt) (Fig. 4*C*) by Western blot analysis. Among the 3 major subfamilies of MAPKs, phosphorylated p38 (p-p38) levels did not change significantly, but phosphorylated ERK (p-ERK) and phosphorylated JNK (p-JNK) demonstrated a significant increase upon RANKL stimulation, and matrine treatment largely inhibited their phosphorylation upon RANKL stimulation. These results indicate that matrine can inhibit RANKL-induced activation of MAPKs and the AKT pathway in osteoclasts.

### Matrine suppresses osteoclastogenesis-related genes expression

**Figure 5.**
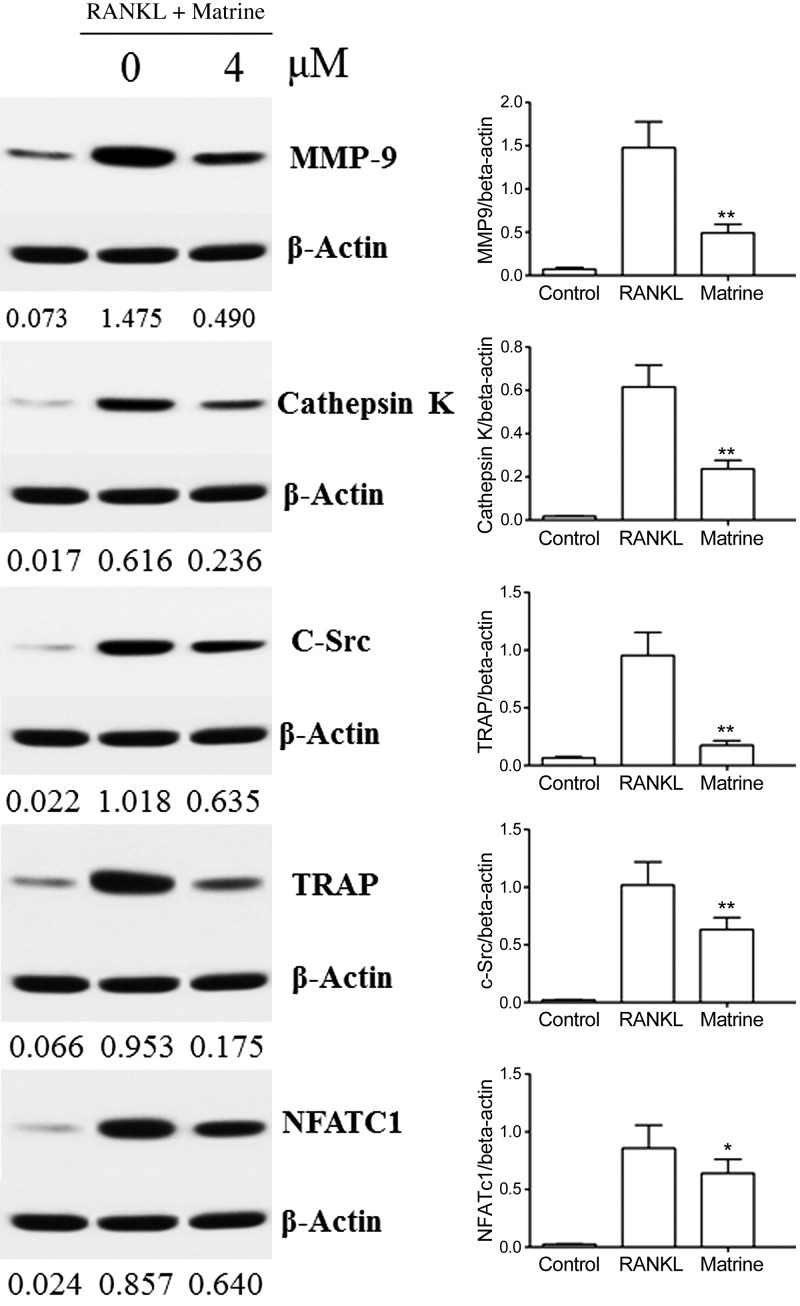
Matrine suppresses osteoclastogenesis-related marker gene expression. Matrine-inhibited protein expression levels of MMP-9, cathepsin K, C-Src, TRAP, and NFATc1 in RAW264.7 cells.

Osteoclast differentiation is completed by the expression of a large number of related marker genes, such as MMP-9, NFATc1, TRAP, C-Src, and cathepsin K, most of which are target genes of NFATc1 (21). NFATc1 is a well-known master regulator of osteoclastogenesis and function. In this experiment, RAW264.7 cells were divided into 3 groups: a control group, RAW264.7 cells induced with RANKL, and RAW264.7 cells induced with RANKL and treated with matrine. The results suggest that matrine can suppress the NF-κB, MAPK, and AKT pathways and NFATc1 expression. We also investigated whether matrine regulates osteoclastogenesis-related marker gene expression. Our results indicate that matrine can inhibit RANKL-induced protein levels of MMP-9, cathepsin K, C-Src, TRAP, and NFATc1 (**Fig. 5**).

### Effects of matrine on bone loss and osteoclast activity in OVX mice

**Figure 6.**
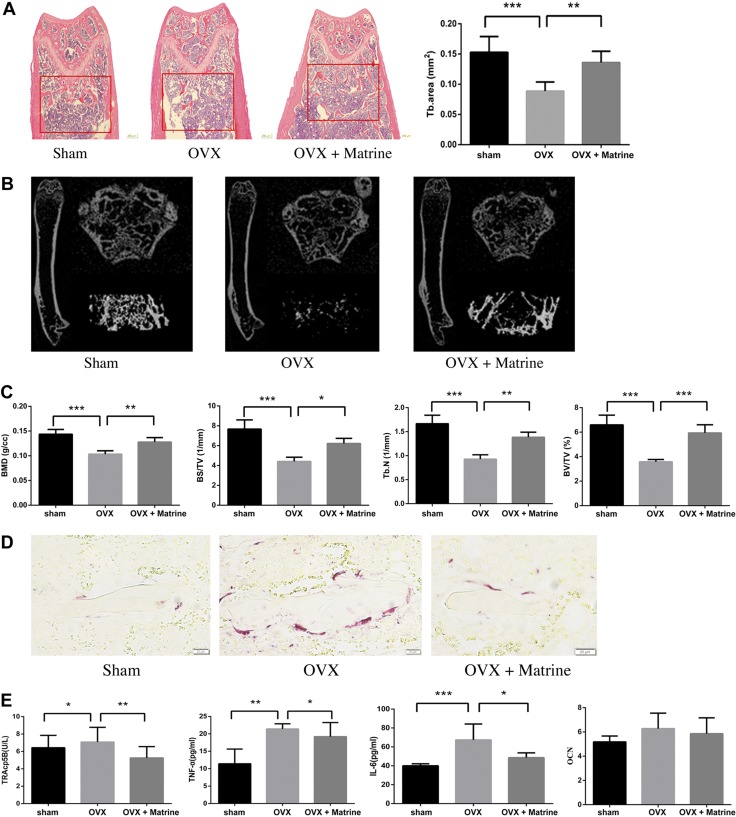
Matrine inhibits ovariectomy-induced bone loss *in vivo*. *A*) Representative H&E staining of femoral sections and difference of trabecular area from each group 6 wk after the operation. *B*) Representative micro–computed tomography sections of femur from sham-treated, OVX, and matrine-treated OVX (OVX + matrine) mice. *C*) Trabecular number (Tb.N), BS/TV, BV/TV, and BMD were analyzed. *D*) Representative TRAP-stained histologic femur sections of the long bone from sham-treated, OVX, and matrine-treated OVX mice. *E*) IL-6, TNF-α, TRAcp5B, and OCN were examined in serum. *P < 0.05, **P < 0.01, ***P < 0.001.

We evaluated the effects of matrine on bone loss using an OVX mouse model. The result showed that at 6 wk after the operation, OVX mice exhibited a significant loss of trabecular bone, represented by decreased BMD, trabecular BV/TV, trabecular BS/TV, and trabecular number, compared with sham-treated mice. Treatment with matrine in OVX mice markedly inhibited trabecular bone loss, as shown by H&E stain, compared with OVX mice treated with normal saline (**Fig. 6*A***). These findings were further corroborated by microcomputed tomography. The 2- and 3-dimensional structure as measured by trabecular BV/TV, BS/TV, trabecular number, and BMD are shown in Fig. 6*B*, *C*.

We examined whether matrine prevented ovariectomy-induced bone loss by inhibiting osteoclast differentiation. Matrine significantly increased the TRAP-positive cells in femur (Fig. 6*D*). Compared with OVX mice, matrine-treated OVX mice displayed decreased serum IL-6, TNF-α, and TRAcp5B (*P* < 0.05), but OCN was not altered significantly (*P* > 0.05), which suggested that matrine inhibited the bone loss in OVX mice through inhibiting osteoclastogenesis rather than by promoting osteogenesis *in vivo* (Fig. 6*E*).

## DISCUSSION

**Figure 7.**
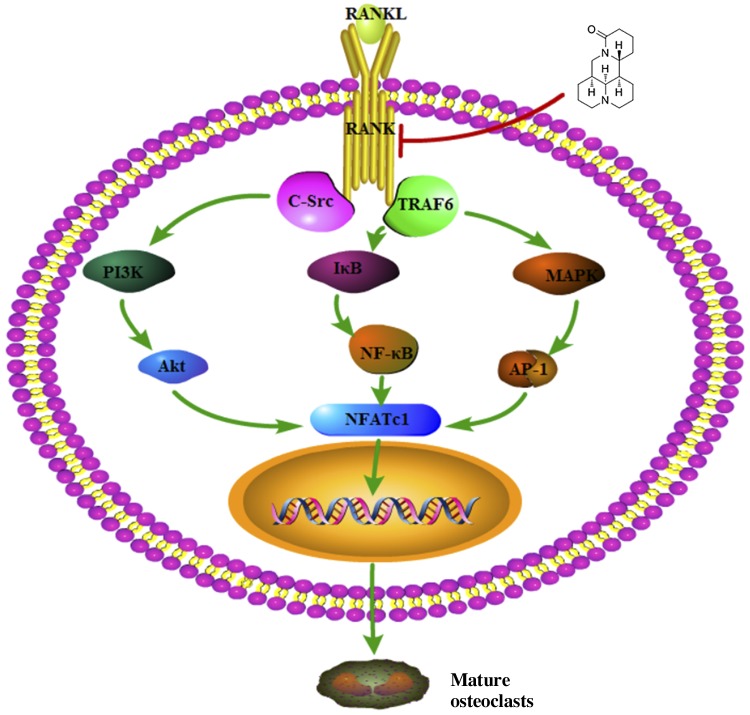
Matrine inhibits osteoclastogenesis through multiple pathways of RANKL signaling.

In this study, we showed that matrine significantly prevented bone loss and inhibited osteoclastogenesis *in vitro* and *in vivo*. This is the first study to demonstrate the effects of matrine on ovariectomy-induced osteoporosis and bone metabolism. As for the molecular mechanisms, multiple pathways, including NF-κB, MAPKs, and AKT, the downstream pathways of RANKL activated during osteoclastogenesis, were significantly inhibited by matrine (**Fig. 7**). Matrine has a very good effect on decreased bone loss, and, based on the matrine structure, we can further design various ramifications for better antiosteoporotic activities.

PMOP is the most common form of primary osteoporosis. PMOP leads to increased risk of osteoporotic fractures (22) and poses a great threat to older women (23). During the pathogenesis of PMOP, overactivated osteoclastogenesis with excessive bone resorption is one important factor (24). Thus, inhibiting osteoclast differentiation remains an important strategy for PMOP treatment (25). Regarding osteoclastogenesis, a growing understanding of bone remodeling indicates that inflammation significantly contributes to the pathogenesis of osteoporosis (26). After the withdrawal of estrogen, a potent inflammation inhibitor (27–29), the elevated proinflammatory cytokines (mainly TNF-α and IL-6) serve as primary mediators of the accelerated bone loss at menopause (30–33). These proinflammatory cytokines increased RANK expression in osteoclast precursor cells and increased RANKL expression in lymphocytes, mesenchymal stem cells, and osteoblasts (34). Thus, inhibiting inflammation provides an option for PMOP prevention and treatment (35).

Many active drug monomers show inhibitory effects on osteoclastogenesis (11, 36–40). Matrine is an alkaloid found in plants of the *Sophora* genus. It has a variety of pharmacological effects, including anti-inflammation effects, and has been widely used clinically with good effects in the treatment of liver fibrosis (41). It has been demonstrated that matrine exhibited protective effects on LPS-induced acute lung injury (42) and focal cerebral ischemia (43) by inhibiting the inflammatory response. It mainly inhibited NF-κB activation, which shares the pathway for osteoclastogenesis (44). Thus, theoretically, matrine is a possible osteoprotective agent.

We carried out this study to explore the effects of matrine on PMOP as well as the molecular mechanisms. *In vivo*, we found that matrine significantly prevented bone loss at 6 wk after ovariectomy in mice, as shown by H&E staining of the distal femur and microcomputed tomography. For TRAP staining, we found that matrine significantly reduced the number of activated osteoclasts around the trabecula. The serum level of TRAcp5B was also significantly reduced. *In vitro*, matrine significantly inhibited osteoclast differentiation in RAW 264.7 cells and BMMs induced by RANKL and M-CSF demonstrated by TRAP staining. F-actin ring formation was also significantly inhibited by matrine. Therefore, we speculated that matrine prevented ovariectomy-induced bone loss through inhibiting osteoclastogenesis.

RANKL is essential for osteoclastognesis (45). After RANKL combines with RANK on osteoclast precursor cells, the downstream pathways are activated, during which NF-κB, MAPKs, and AKT play a major role in signal transduction. The NF-κB family includes p105 (NF-κB1), p100 (NF-κB2), RelA (P65), RelB, and Relc (46). After RANK is activated by RANKL, p105 is processed to P50 constitutively and forms dimers usually with RelA. P50/P65 dimers are translocated to the nucleus for gene transcription. Previous studies have demonstrated that epoxyeicosanoids inhibited osteoclastogenesis through modulation of multiple pathways both upstream and downstream of RANKL signaling (11). Caffeic acid suppresses osteoclastogenesis and bone loss through inhibiting RANKL-induced MAPKs and Ca2þ-NFATc1 signaling pathways (38). In this study, we found that matrine prevented translocation of P65 from cytoplasm to nucleus showed by confocal fluorescence in RAW264.7 cells after RANKL/M-CSF induction. Western blot showed that matrine significantly reduced P65 and P50 phosphorylation in a time-dependent manner compared with the vehicle group. Matrine could block the canonical NF-κB pathway.

MAPKs and PI3/AKT are also involved in RANK downstream cell signaling transduction and osteoclastogenesis (45). In response to RANKL, RANK interacts with TRAFs, among which TRAF6 is crucial for activating MAPKs (47). TRAF6 activates the TAB1/TAB2/TAK1 complex, which leads to the activation of IKK-β and MAPKs (48). RANK also activates Src family kinase signaling, which leads to AKT activation through interactions between TRAF6 and Cbl scaffolding proteins (49). In this study, matrine inhibited phosphorylation of ERK, JNK, P38, and C-fos in the MAPK pathway and inhibited AKT activation, which led to decreased expression of osteoclastogenesis-related markers, including MMP-9, TRAP, C-Src, and cathepsin K.

Limitations within our study that indicate the need for future work. First, *in vivo* studies proved that matrine could significantly prevent bone loss and inhibit osteoclast activation. However, it raises the question of what effects matrine has on osteogenesis and bone formation. Because there was no statistically significant difference in serum OCN levels between groups, we speculated that matrine had no significant effects on osteogenesis, and mainly inhibited osteoclastogenesis. Second, the cellular signaling mechanisms involving modulation of signal transduction pathways have yet to be elucidated. Matrine could inhibit MAPKs, NF-κB, and AKT pathways, indicating that matrine could probably influence the upstream of intracellular signal transduction during osteoclastogenesis. Third, the interaction target of matrine has not been clarified and will be addressed in future studies. Matrine has many problems, such as poor bioavailability, high toxicity, and poor solubility in water. Thus, based on matrine chemical structure, various derivatives could be synthesized to search for more effective drugs. Because matrine shows a significant inhibitory effect on osteoclastogenesis, it probably has therapeutic effects on various osteoclast-related diseases (*e.g.*, rheumatoid arthritis and bone-metastasized tumors); this needs further verification.

In summary, our findings demonstrate that matrine could serve as a novel inhibitor of osteoclastogenesis by suppressing multiple signaling pathways. It is of great significance to determine whether matrine can be used as a beneficial alternative preventive and therapeutic option for osteoclast-related disorders.

## Abbreviations

BMD: bone mineral density
BMM: bone marrow monocyte
BS/TV: bone surface area/total value
BV/TV: bone value/total value
H&E: hematoxylin and eosin
MMP-9: matrix metalloproteinase 9
OCN: osteocalcin
OVX: ovariectomized
PMOP: postmenopausal osteoporosis
RANKL: receptor activator for NF-κB ligand
TRAF: TNF receptor–associated factor
TRAP: tartrate-resistant acid phosphatase
